# 28160 Evaluation of women's empowerment in a community-based HPV self-sampling social entrepreneurship in Peru: Mixed-method study

**DOI:** 10.1017/cts.2021.605

**Published:** 2021-03-30

**Authors:** Michelle B. Shin, Mary Elizabeth Dotson, Maria Valderrama, Marina Chiappe, Ruanne V. Barnabas, Kristjana Asbjornsdottir, Sarah Iribarren, Sarah Gimbel, Patricia J. Garcia

**Affiliations:** 1 University of Washington School of Nursing; 2 Duke University Department of Biomedical Engineering and the Duke Global Health Institute; 3 School of Public Health Universidad Peruana Cayetano Heredia; 4 University of Washington Department of Global Health

## Abstract

ABSTRACT IMPACT: Understanding the women community leaders’ sense of relational and financial empowerment in the social entrepreneurship context will be key to developing a sustainable pathway to scale-up community-based HPV self-sampling programs in low resource settings. OBJECTIVES/GOALS: The Hope Project, a social entrepreneurship (SE) near Lima, Peru, trains women leaders (Hope Ladies) to promote human papillomavirus (HPV) self-sampling in their communities. This study aims to evaluate the Hope Ladies’ own relational/financial empowerment after participating in the program. METHODS/STUDY POPULATION: The Hope Ladies participated in semi-structured in-depth interviews (n= 9) and 8-question 5-point Likert-scale survey (n=16) that evaluated their relational/financial empowerment after participating in the social entrepreneurship. The interview and the survey questions were developed using validated empowerment frameworks, indicators, and theory, respectively: 1) Kabeer’s conceptual framework, 2) International Center for Research on Women (ICRW), and 3) Relational Leadership Theory (RLT). Direct content analysis was used to deductively evaluate the interviews with predetermined codes and categories of empowerment. Descriptive statistics were used to analyze the survey results. RESULTS/ANTICIPATED RESULTS: All reported experiencing empowerment in the SE. Interviews: The codes were mapped onto 3 categories/9 sub-categories: 1) voicing confidence (willingness to challenge social/gender norms); 2) social resources (new skills, knowledge, self-efficacy, access to networks, role models); 3) financial gains (helpful but not the primary motivation to continue as Hope Ladies, and not enough to override traditional household roles/priorities). Survey: 75% indicated an increase in social contacts, confidence in discussing reproductive topics (75%), comfort with medical facilities (44%), ability to help the community (62.5%), and ability to make household purchasing decisions (36%) since joining the program. DISCUSSION/SIGNIFICANCE OF FINDINGS: The Hope Ladies’ experience in this SE demonstrated the complex relationship between various domains of empowerment (e.g., relational/financial). More studies are needed to elucidate the relationship between empowerment and worker retention/performance to inform scale-up of HPV self-sampling SE’s.

